# Xingnao Jiutan tablets modulate gut microbiota and gut microbiota metabolism to alleviate cerebral ischemia/reperfusion injury

**DOI:** 10.3389/fcimb.2024.1497563

**Published:** 2025-02-20

**Authors:** Yanyan Chen, Jing Zhang, Xiaoran Hou, Shijiao Cai, Jingyue Zhang, Yidan Gou, Hanxu Zhang, Yang Zhai, Hengjie Yuan

**Affiliations:** Department of Pharmacy, Tianjin Medical University General Hospital, Tianjin, China

**Keywords:** Xingnao Jiutan tablets, cerebral ischemia/reperfusion, gut microbiota, metabolism, brain-gut axis

## Abstract

**Introduction:**

Xingnao Jiutan tablets (XNJT), a compound Chinese medicine, have been applied to the treatment of the sequelae of cerebral thrombosis or cerebral hemorrhage, transient cerebral ischemia, and central retinal vein obstruction, etc., but the underlying mechanisms are not yet clear. This research focused on examining the impact of XNJT for cerebral ischemia/reperfusion (MCAO/R) injury, utilizing gut microbiota and metabolomic studies.

**Methods:**

The primary components of XNJT were identified through the application of the HPLC technique. We established a MCAO/ R model in mice and conducted behavioral evaluations, cerebral blood flow measurements, and TTC staining. We used ELISA, high-throughput 16S rDNA gene sequencing, and metabolomics techniques to detect inflammatory factors, microbial populations, and metabolites, respectively. Finally, we performed Spearman correlation analysis to investigate the relationships among gut microbiota and metabolites, comprehensively exploring the mechanisms of XNJT to alleviate cerebral ischemia-reperfusion injury.

**Results:**

We discovered that XNJT effectively enhanced neurological performance, alleviated cerebral infarction, diminished neuronal cell death, and increased cerebral blood flow. Moreover, XNJT downregulated the secretion of pro-inflammatory cytokines like TNF, IL-6, and IL-1b. Additionally, XNJT improved gut microbiota levels in MCAO/R mice, particularly *Bacteroides*, *Firmicutes, Escherichia-Shigella*, and *Ligilactobacillus*. Furthermore, XNJT primarily modulated differential metabolites in the gut through Glycerophospholipid, Linoleic acid, and Sphingolipid metabolism pathways. Spearman correlation analysis revealed significant associations among intestinal microbiota and various metabolites.

**Discussion:**

In summary, our findings suggest that XNJT can improve cerebral ischemia/reperfusion injury outcomes, reduce inflammatory responses, and regulate gut microbiota and differential metabolites. It’s possible that the potential mechanisms are connected to controlling gut microbiota and metabolism.

## 1 Introduction

Stroke, a disorder of the central nervous system, is marked by high morbidity, mortality, disability, and economic burden due to brain tissue damage from blocked or burst blood vessels in the brain (Grysiewicz et al., 2008). Research indicates that worldwide, stroke ranks as the second most common cause of mortality, representing 11.6% of total deaths, and is the third leading reason for disability (Demsie and Lorkowski, 2020; Feigin et al., 2022). Additionally, China has the highest stroke prevalence worldwide, with a trend toward younger patients (Zhou et al., 2019; Xian et al., 2022). Stroke types include ischemic stroke and cerebral hemorrhagic stroke, where ischemic stroke occurs more frequently, constituting 60% to 80% of all stroke cases (Herpich and Rincon, 2020; Liu et al., 2020). The primary cause of ischemic stroke is the blockage of the intracranial artery, leading to intricate brain neuropathological alterations such as excitotoxicity, oxidative stress, neuroinflammation, and breakdown of the blood-brain barrier, culminating in brain tissue death and neurological impairments (Maida et al., 2020; Candelario-Jalil et al., 2022; Huang et al., 2022; Rajeev et al., 2022; Zong et al., 2022). Clinical manifestations of neurological deficits include hemiparesis, speech difficulties, facial asymmetry, and impaired consciousness. Presently, thrombolysis and interventional treatment stand as the most efficacious therapies, yet they come with high costs and constraints like a limited time frame and related risks. Therefore, it’s critically important to develop efficient and economical treatment methods to reduce the burden of strokes on both society and families. In conclusion, studying the pathophysiologic process of ischemic stroke and establishing a comprehensive system for its prevention, diagnosis, treatment, and prognosis is of great significance.

A growing collection of studies suggests that ischemic stroke affects gut microbiota and that regulating gut flora could be an innovative approach to preventing and treating strokes (Pluta et al., 2021; Yamashiro et al., 2021; Zhang et al., 2021). Dysbiosis of the gut microbiota can disrupt brain function and result in neurological disorders (Durgan et al., 2019). Furthermore, gut microbiota-related metabolites, including short-chain fatty acids and lipopolysaccharides, show a significant correlation with the development and outcomes of stroke (Chen et al., 2019b; Cheng et al., 2022). This suggests a role for the brain-microbe-gut axis in the pathology of strokes. Gut microbiota serves as a “two-way” communication system between the gastrointestinal system and the brain.

**Figure 1:**
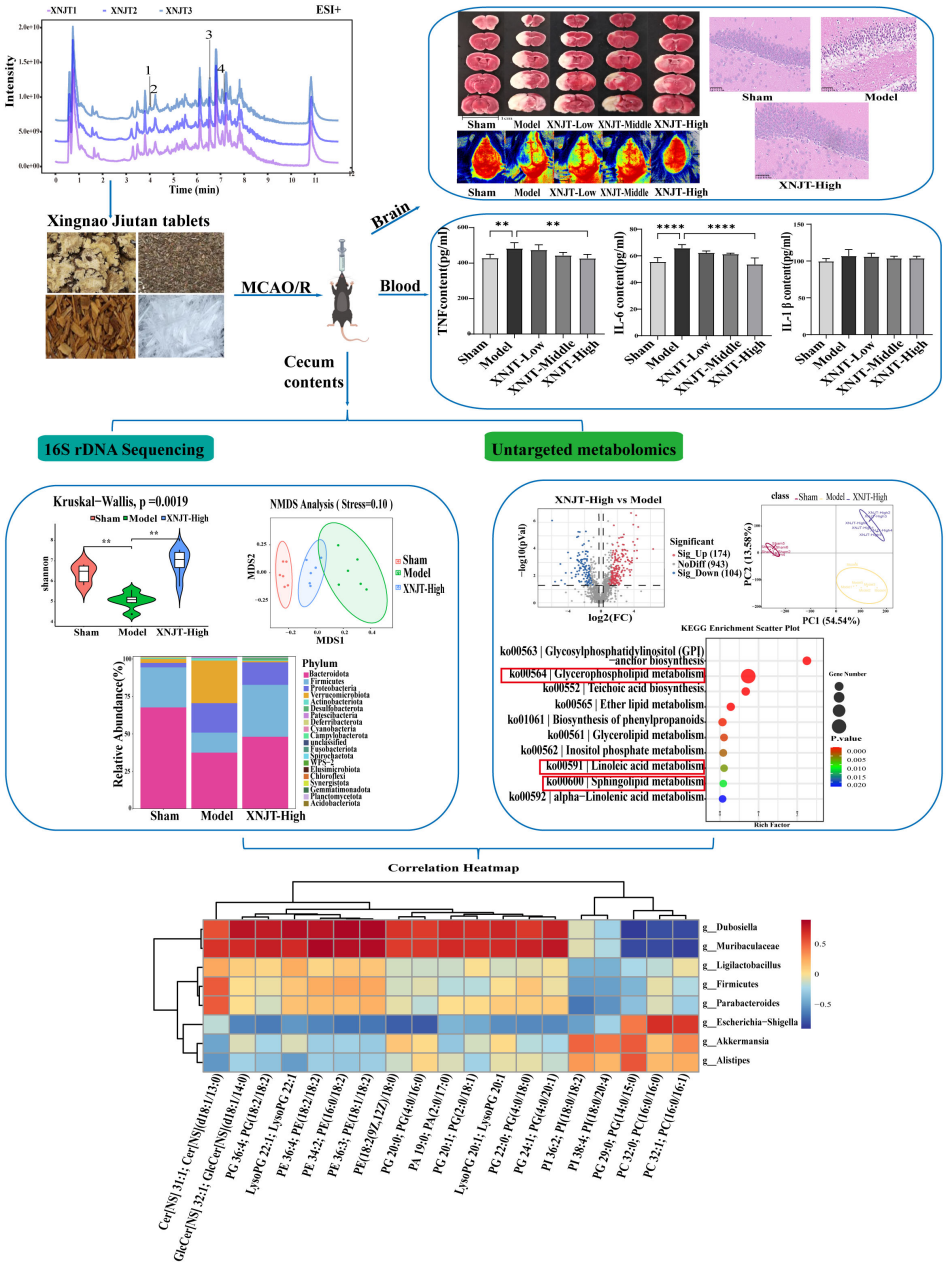
Graphical abstract of this study. **p <0.01, ****p <0.0001.

Recently, the healing impact of Chinese medicine on a range of illnesses has gained broad acknowledgment, along with its recognized promise in medical care (Zhai et al., 2023). Chinese medications, characterized by their diverse components, extensive coverage, and multiple targets, are vital in stroke treatment (Chang et al., 2016). Research indicates that traditional Chinese medicinal practices have the potential to alter gut microbiota and its by-products (Feng et al., 2018; Gong et al., 2020). XNJT, a hospital preparation from Tianjin Medical University General Hospital, can activate blood circulation, remove blood stasis, and promote overall circulation It is used to treat the sequelae of cerebral thrombosis or cerebral hemorrhage, transient cerebral ischemia, and central retinal vein obstruction. XNJT is mainly made of 4 kinds of Chinese herbs, *Ligusticum striatum* DC. (Chuan Xiong), *Leonurus japonicus* Houtt. (Chao Chong Wei Zi), *Santalum album* L. (Tan Xiang), and *Dryobalanops aromatica* C.F.Gaertn. (Bing Pian). In the clinic, XNJT has the potential to markedly enhance the clinical manifestations in patients with sequelae of cerebral infarction, patients with the drug after the cerebral vascular resistance is reduced, the blood flow changes, proving that the drug helps to improve the cerebral blood supply insufficiency (Xie and Tang, 2005). The study by Xie and Tang assessed 100 patients with cerebral infarction, encompassing measurements of carotid blood flow velocity, blood flow volume, cerebrovascular resistance, and changes in blood rheology, with an overall efficacy rate of 72%. In Chinese medicine, *Ligusticum striatum* DC., is extensively employed in the treatment of cardiovascular and neurovascular diseases. Ferulic acid, a key component of *Ligusticum striatum* DC., is known to reduce memory impairments and offer defense against oxidative stress and apoptosis caused by brain ischemia/reperfusion injury (Ren et al., 2017; Liu et al., 2022). Numerous studies on animals have demonstrated that tetramethylpyrazine reduces infarct size, neurological scores, and cerebral edema in models of permanent and transient cerebral ischemic injury (Kao et al., 2013; Xiao et al., 2013; Tan et al., 2015). *Leonurus japonicus* Houtt., made from Motherwort Fruit, is known for its ability to invigorate blood circulation, remove blood stasis, purify the liver, and brighten the eyes. Leonurine, a major component of Leonurus japonicus Houtt., reduced the reactive oxygen species concentrations in mitochondria extracted from the ischemic cortex (Qi et al., 2010). *Santalum album* L. enhances the activity of antioxidant enzymes and scavenges oxygen free radicals (Xu et al., 2022). *Dryobalanops aromatica* C.F.Gaertn. inhibits inflammatory factors, reduces oxidative stress, and maintains brain function (Ma et al., 2023). Importantly, some botanical drugs in XNJT, such as *Santalum album* L., have been shown to modulate the intestinal microbiota. Investigating if XNJT’s anti-stroke properties are partially due to its influence on gut microbiota and metabolites is valuable. Therefore, our research focused on examining how XNJT influences gut microbiota and its metabolites in mice suffering from middle cerebral artery occlusion/reperfusion (MCAO/R), employing 16S rDNA gene sequencing combined with untargeted metabolomics analysis (**Figure 1**).

## 2 Materials and methods

### 2.1 Reagents and chemicals

XNJT was provided by Tianjin Medical University General Hospital. The approval number was Z2007590. The details of the XNJT employed in this research are described in **Table 1**. The chemical profile follows the standards set forth in the ConPhyMP statement (Heinrich et al., 2022). Sodium carboxymethyl cellulose (CMC-Na, C8621), 4% paraformaldehyde (P1110), hematoxylin-eosin stain kit (G1120), and TTC Solution (2%, G3005) were purchased from Solarbio (Beijing, China). The mouse TNF (YJ002095), IL-6 (YJ063159), and IL-1β (YJ098416) ELISA kit were provided by Enzyme-linked Biotechnology (Shanghai, China). Monofilament (0621) for MCAO/R was purchased by Yushun Biotech (Henan, China). Botanical names are referenced from “World Flora Online”, http://www.worldfloraonline.org.

**Table 1:** XNJT composition.

Botanical name	Chinese name	Medicinal Part	Grams, g
*Ligusticum striatum* DC.	Chuan Xiong	Rhizome	255.3
*Leonurus japonicus* Houtt.	Chao Chong Wei Zi	Fruit	425.6
*Santalum album* L.	Tan Xiang	Heartwood	212.8
*Dryobalanops aromatica* C.F.Gaertn.	Bing Pian	Leaf and bark extracted Crystalline	6.4

### 2.2 XNJT preparation method and fingerprint analysis

The primary procedural stages of XNJT included: grind the half amount of *Ligusticum striatum* DC. and *Santalum album* L. into fine powder. Take the other half amount of *Ligusticum striatum* DC., *Santalum album* L., and *Leonurus japonicus* Houtt., and boil them in water twice. The first boiling should last for 2 hours, and the second boiling for 1.5 hours. Combine the decoctions, filter them, and evaporate the filtrate under reduced pressure until the relative density reaches 1.36-1.38 (at 60°C), forming a clear paste. Mix the paste with the aforementioned fine powder, dry it, and crush it into fine particles. Add an appropriate amount of powdered sugar, granulate it, dry it, and spray it onto *Dryobalanops aromatica* C.F.Gaertn. dissolved in a suitable amount of ethanol. Mix well, press it into 1000 tablets, and coat them with sugar shell.The daily dose for an adult in this study was 4.86g/70 kg/d.

The analysis of LC-MS/MS utilized a Vanquish (Thermo Fisher Scientific, USA) high performance liquid chromatography (HPLC) system, equipped with a Phenomenex Kinetex C18 (2.1 mm × 100 mm, 2.6 μm) linked to the Orbitrap Exp loris 120 mass spectrometer (Orbitrap MS, Thermo). Solvent A (0.01% acetic acid in water, v/v) and solvent B (isopropanol and acetonitrile, 1:1, v/v) were used for gradient elution, and the injection volume was 2 μL. The MS/MS spectral analysis was conducted utilizing an Orbitrap Exploris 120 mass spectrometer. Settings for mass spectrometry included: a sheath gas flow rate of 50 Arb, an Aux gas flow rate of 15 Arb, a capillary temperature of 320°C, a complete MS resolution of 60000, a MS/MS resolution of 15000, collision energy of SNCE 20/30/40, and spray voltages of 3.8 kV (positive) and -3.4 kV (negative) respectively. ProteoWizard was employed to transform the unprocessed data into the mzXML format. Additionally, identifying metabolites through R software and XCMS-based methods for detecting peaks, extracting, aligning, and integrating. For identifying metabolites, the R software along with BiotreeDB (V3.0) were utilized. The database used to identify metabolites was BiotreeDB (V3.0).

### 2.3 Animals

Male C57BL/6J mice, aged 8 weeks and weighing 19-21g, were acquired from Beijing Vital River Laboratory Animal Technology Co., Ltd (SCXK (Jing) 2021-0006). Before the experiment, the animals resided in a designated specified pathogen-free (SPF) animal room at the Institute of Neurology, General Hospital of Tianjin Medical University, and acclimated for 1 week. The mice were kept under regulated environment of 22-25°C and 60 ± 5% humidity, with unrestricted access to both food and water. The procedure for the experiment adhered to the guidelines governing the treatment of experimental animals. (approval number IRB2023-DWFL-007).

### 2.4 Animals experimental design

The mice were arbitrarily segmented into five distinct groups: sham group (Sham, n = 6), MCAO/R group (Model, n = 6), low dose group (XNJT-Low, n = 6), middle dose group (XNJT-Middle, n = 6), and high dose group (XNJT-High, n = 6). The low, medium, and high doses for mice were 0.6318, 1.2636, and 2.5272 g/kg/d, respectively, which were 1, 2, and 4 times the daily dose for an adult. XNJT were ground into powder and dissolved in 0.5% CMC-Na (C8621, Solarbio,Beijing, China) before gavage. In the XNJT-Low, XNJT-Middle, and XNJT-High groups, mice received daily gavage with XNJT solution (0.6318g/kg/d), XNJT solution (1.2636g/kg/d), or XNJT solution (2.5272 g/kg/d) for seven days pre-surgery, respectively. Equal volumes of 0.5%CMC-Na were provided through gavage in both Sham and Model groups.

Conducted an hour subsequent to the final administration, the MCAO procedure adhered to a protocol already in place (Xu et al., 2021). According to the Zea-Longa method, mice underwent a 12-hour fast prior to surgery, with free access to water only (Longa EZ et al., 1989; Ying Huang et al., 2022). In summary, the animals were anesthetized using isoflurane and kept in a supine position. A cut was made along the neck’s central line to expose the common carotid artery (CCA), external carotid artery (ECA), and internal carotid artery (ICA). To avert bleeding, the CCA and ECA were secured near the end using 5-0 nylon stitches, the CCA was loosely fastened at the end, and the ICA was tied using a microarterial clip. A minor cut was created in the CCA, followed by the insertion of a monofilament into the ICA until resistance was detected, after which it was halted. A 3-0 suture was used to fasten the monofilament and seal the cut. Following an hour of ischemia, the monofilament was methodically extracted to facilitate reperfusion. The Sham group experienced an identical process, with the exception of inserting monofilaments. After surgery, the mice were moved to an electric blanket maintained at 37 ± 0.5°C until they regained consciousness.

### 2.5 Evaluation of neurological score

Neurological function scoring was performed using the Zea-Longa method (Longa EZ et al., 1989). Each group’s neurological capabilities were evaluated 24 hours after ischemia/reperfusion, with a total score of 4 points (Yang et al., 2022a; Zhang et al., 2022). No neurological deficit scored 0 points; the inability to fully extend the front paw on the paralyzed side scored 1 point; turning to the paralyzed side when walking scored 2 points; tilting to the paralyzed side when walking scored 3 points; and the inability to walk automatically and loss of consciousness scored 4 points.

### 2.6 Cerebral blood flow detection

Mice were anesthetized and their skin prepared. The head was fixed with a stereotaxic apparatus and the skull exposed. The flow of blood in the brain was tracked within a minute through the application of laser speckle contrast analysis technology. The detection values of the relatively stable period (30s) in the surgical area were selected by PIMSoft for statistical analysis.

### 2.7 Sample collection and preparation

Mice were anesthetized after neurological behavioral scoring, and blood samples were collected. Samples of blood were left undisturbed for an hour, followed by centrifugation at 3000 rpm for a duration of 10 minutes, and the clear liquid above the sediment was preserved at -80°C. The brains were removed, with those used for TTC staining refrigerated at a temperature of -20°C and those for HE staining fixed in 4% paraformaldehyde. The cecum’s contents were extracted, swiftly frozen using liquid nitrogen and maintained at a temperature of -80°C.

### 2.8 Triphenyltetrazolium chloride staining

The brains of the mice were preserved in a fridge at -20°C for a duration of 20 minutes, then cut into 5 slices, each 2 mm thick. The slices were placed in 2% TTC solution for a duration of 30 minutes at a temperature of 37°C, ensuring even staining by turning them periodically (Benedek et al., 2006). Subsequently, they underwent fixation in 4% paraformaldehyde for a duration of 2 hours and were photographed. Infarcted tissue volume was measured using Image J software for determining the volume of the infarct. The infarct rate (%) was determined by the formula: infarct area/whole brain area × 100%.

### 2.9 Hematoxylin - eosin staining

The brain and intestinal tissues underwent fixation in 4% paraformaldehyde for a duration of 24-48 hours, followed by dehydration using 75% ethanol, 85% ethanol, 90% ethanol, 95% ethanol, 100% ethanol I, 100% ethanol II sequentially, clarification in xylene, encasement in paraffin, and slicing. The samples underwent deparaffinization using xylene followed by rehydration in progressively lower ethanol levels. The samples underwent a 3-minute hematoxylin staining, were treated with hydrochloric acid to revert to blue, and followed by a 2 seconds eosin staining process. The sections were cleared in xylene and sealed to observe morphological changes in brain and intestinal tissue.

### 2.10 Enzyme linked immunosorbent assay

The concentrations of tumor necrosis factor (TNF) (Grimstad, 2016), interleukin-1β (IL-1β), and interleukin-6 (IL-6) in the serum were quantified utilizing ELISA kits. First, allow the ELISA kits to equilibrate at room temperature for 20 minutes. Subsequently, add 50 μL of standards at varying concentrations to the designated wells. For the sample wells, introduce 10 μL of the sample to be tested followed by 40 μL of sample diluent. Except for the blank wells, to both the standard and sample wells, add 100 μL of horseradish peroxidase (HRP)-conjugated detection antibody, seal with an adhesive cover, and incubate at 37°C for 60 minutes in a temperature-controlled chamber. After discarding the liquid, pat dry on absorbent paper, fill each well with wash buffer, let it stand for 1 minute, decant the wash buffer, and pat dry again. Repeat this washing process five times. Next, add 50 μL each of substrates A and B to each well and incubate at 37°C in the dark for 15 minutes. Finally, add 50 μL of stop solution to each well, and within 15 minutes, measure the absorbance of each well at a wavelength of 450 nm using an enzyme labeling instrument.

### 2.11 16S rDNA gene sequencing

16S rDNA gene sequencing of cecum contents was executed by LC-Bio (Zhejiang, China). Total microbial DNA was isolated from each specimen using the CTAB technique, with the quality of the DNA verified through agarose gel electrophoresis. Amplification of the 16S rDNA sequences was achieved through PCR, utilizing primers specific to V3-V4 (341F, 5’-CCTACGGGNGGCWGCAG-3’; 805R, 5’-GACTACHVGGGTATCTAATCC-3’), and the PCR products were confirmed by 2% agarose gel electrophoresis. PCR outputs underwent purification using AMPure XT beads (Beckman Coulter Genomics, Danvers, MA, USA) and were measured using Qubit (Invitrogen, USA). The assessment of purified PCR products was conducted with the aid of an Agilent 2100 Bioanalyzer (Agilent, USA) and Illumina (Kapa Biosciences, Woburn, MA, USA) for library quantification kits, and the concentration of the qualified libraries should be above 2 nM. Qualified sequencing libraries were pooled after gradient dilution. Subsequently, the mixtures were subjected to sodium hydroxide-induced denaturation to achieve single-stranded DNA for subsequent sequencing procedures. Samples passing the evaluation were sequenced using the NovaSeq 6000 sequencer with 2 × 250 bp paired-end reads. Following sequencing, the paired-end data were processed through demultiplexing, assembly, filtering, and noise reduction to obtain amplicon sequence variants. Alpha diversity analysis, beta diversity analysis, and species annotation were subsequently conducted based on these amplicon sequence variants. Alpha and beta diversity analyses done at the amplicon sequence variants (ASV) level. The reference databases used for taxonomic identification are the SILVA (Release 138, https://www.arb-silva.de/documentation/release138, min confidence:0.7) and NT-16S databases (Release 20230718, min ident:90; min cov:80; max e1e:5).

### 2.12 Untargeted metabolomics analysis

The gathered contents of the cecum were defrosted on ice, followed by the extraction of metabolites using a 50% methanol solution. In summary, 100 mg of the sample underwent extraction using 1 ml of chilled 50% methanol, followed by overnight storage at -20°C. Samples underwent centrifugation at 4000 g for a duration of 20 minutes and were preserved at -80°C for subsequent application. Furthermore, a mixture of QC samples was created using 10 μL of each extract.

First, every chromatographic division was executed utilizing the UltiMate 3000 UPLC system. The process of reversed-phase separation was executed using an ACQUITY UPLC T3 column. The composition of the mobile phase included 5 mM ammonium acetate, 5 mM acetic acid, and solvent B (acetonitrile), keeping the column chamber stable at 40°C. The rate of flow was established at 0.3 milliliters per minute. The conditions for gradient elution included: 0-0.8 minutes, 2% B; 0.8-2.8 minutes, 2% to 70% B; 2.8-5.6 minutes, 70% to 90% B; 5.6-6.4 minutes, 90% to 100% B; 6.4-8.0 minutes, 100% B; 8.0-8.1 minutes, 100% to 2% B; 8.1-10 minutes, 2% B.

The metabolites emerging from the column were identified through a high-resolution tandem mass spectrometer (Q-Exactive), functioning in both positive and negative ion modes. Spectra of metabolite ion precursors (70-1050 m/z) were gathered at a 70000 resolution to attain an AGC of 3e6 and a peak injection duration of 100 ms. The leading three settings for gathering data were configured in DDA mode. The collection of fragment spectra occurred at a 17500 resolution, attaining an AGC between 1e5 and a peak injection duration of 80 ms.To assess the stability of the LC-MS throughout the acquisition, a single quality control sample (mixed sample) was gathered following every 10 samples.

### 2.13 Statistical analysis

Results from the experiments were graphically represented and subjected to statistical examination via ImageJ and GraphPad Prism (version 9.5). Normality was tested using SPSS and if it was normally distributed, the study was tested using one-way ANOVA. If it was non-normally distributed, non-parametric tests were used. A significant difference between groups was considered statistically significant if P ≤ 0.05.

16S rDNA samples were performed on the Illumina NovaSeq platform. The FLASH tool was employed to amalgamate paired-end reads. The raw data underwent quality filtration to identify superior clean tags, adhering to certain filtering parameters as per fqtrim (v0.94). The Vsearch software (v2.3.4) was used to sift through chimeric sequences. The prevalence of features was standardized based on the comparative prevalence of each specimen as per the SILVA (release 138) classification system. Additional graphical representations were created utilizing the R package (v3.5.2). Comparisons between multiple groups with biologically replicated samples were conducted employing the Kruskal-Wallis test, setting a significance level at p<0.05.

Acquired mass spectrometry data were preprocessed using XCMS software. Raw data files from LC-MS were transformed into mzXML format and analyzed using the XCMS, CAMERA, and metaX tools available in R software. Annotation of the metabolites was conducted through the online KEGG and HMDB databases, aligning the precise molecular mass data (m/z) of the samples with the information in these databases. The primary statistical evaluation was conducted using R software (version 4.0), normalizing the proteins’ raw intensity levels by the median. The final significantly different metabolites were identified by meeting three conditions: P<0.05, FC≥1.2, and VIP≥1. The hypergeometric test was utilized to conduct a differential enrichment analysis on KEGG Pathways, identifying functional entries with a notable enrichment of differential proteins at P<0.05.

## 3 Results

### 3.1 XNJT fingerprints

**Figure 2:**
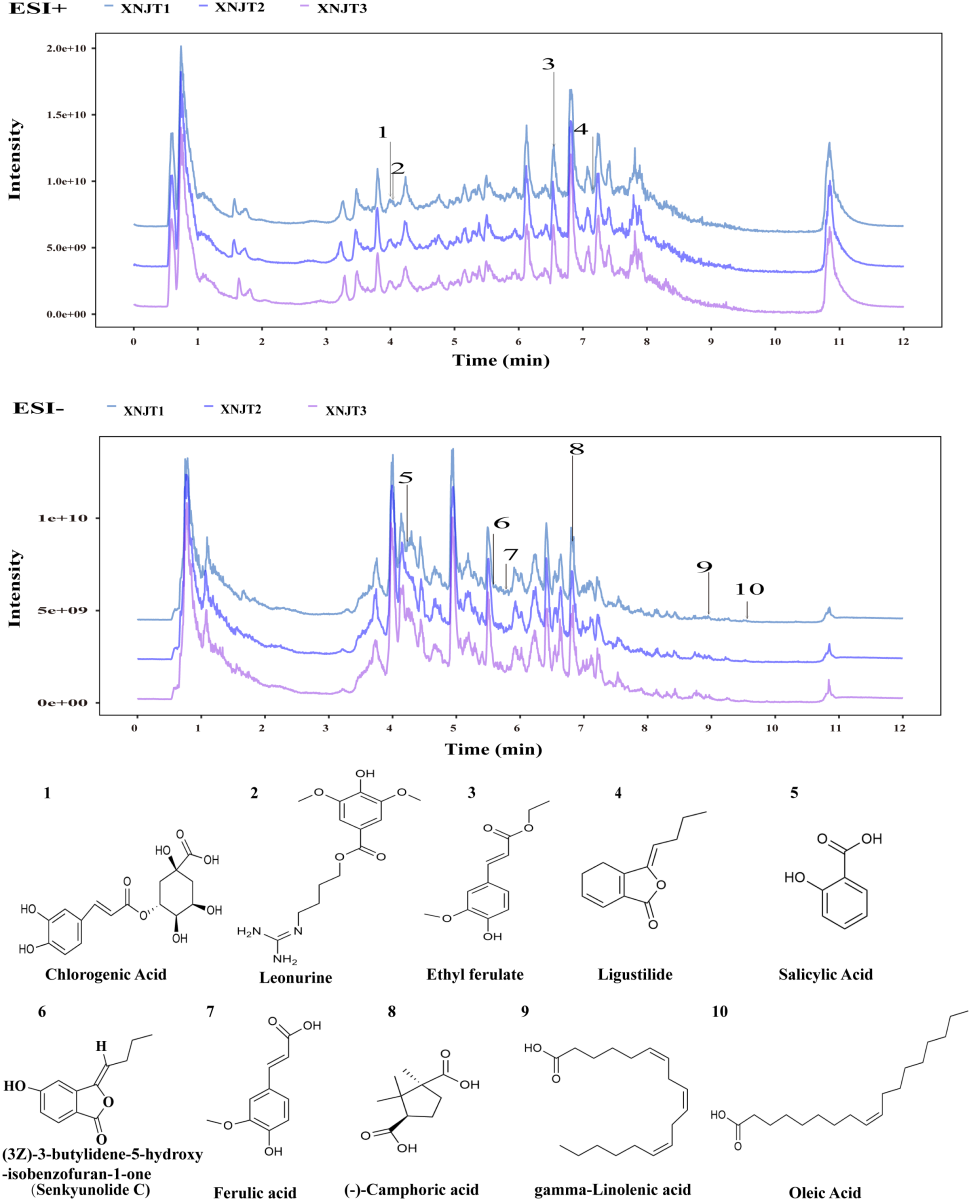
The HPLC chromatogram of XNJT.

**Figure 2** shows the HPLC fingerprints of the XNJT. The comprehensive positive and negative ion chromatograms for XNJT revealed the chemical makeup of every compound. The comprehensive analysis of peak areas and retention times identified 10 constituents in XNJT, specifically ligustilide, trans-ferulic acid, ethyl ferulate, (3Z)-3-butylidene-5-hydroxy-isobenzofuran-1-one (Senkyunolide C), leonurine, salicylic acid, oleic acid, gamma-linolenic acid, chlorogenic acid, and (-)-camphoric acid.

### 3.2 XNJT improves neurological function and attenuates cerebral infarction

**Figure 3:**
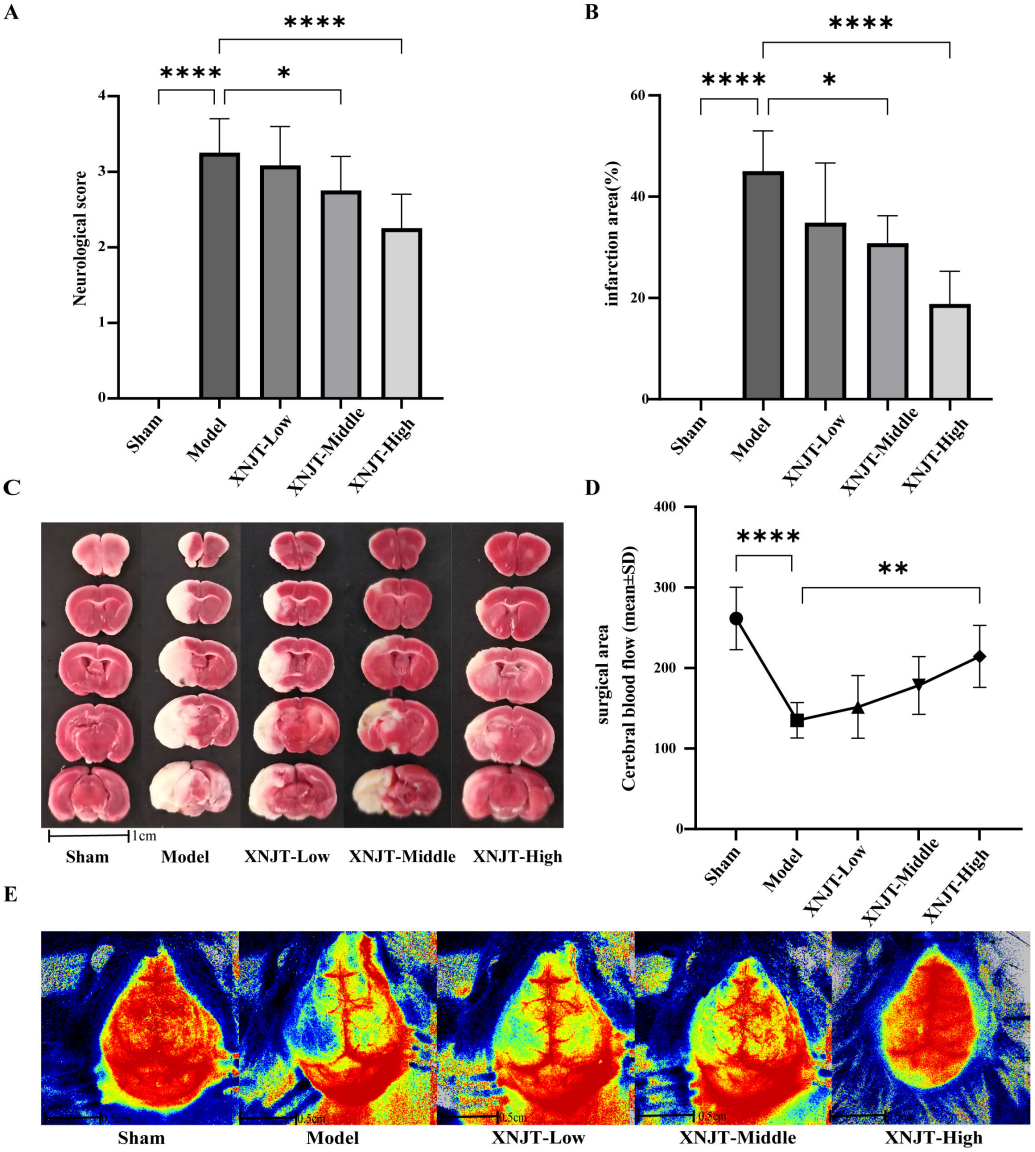
XNJT reduced the neurological deficit score, the infarction area ratio and cerebral blood flow. **(A)** The neurological score (n=12). **(B)** The infarction area(%) (n=6). **(C)** The representative images of TTC staining (n=6). **(D)** Line graph of cerebral blood flow in the surgical area (n=6). **(E)** Representative maps of laser speckle contrast analysis of cerebral blood flow. Statistical differences were examined by one-way ANOVA. *p < 0.05, **p <0.01, ****p <0.0001.

To validate the effect of XNJT on cerebral ischemia/reperfusion, we assessed the neurological deficit score in mice, measured cerebral blood flow, and calculated the cerebral infarct area 24 hours after MCAO/R modeling. The neurologic scores of mice in the Model group were notably higher than those in the Sham group (P<0.0001). Additionally, the neurological scores of mice in the XNJT-Low, XNJT-Middle (P<0.05), and XNJT-High groups (P<0.0001) were lower than those in the Model group, indicating that XNJT reduced brain injury in stroke mice (**Figure 3A**). TTC staining of brain tissue sections revealed no cerebral infarcts in the Sham group and significant cerebral infarcts in the Model group. There was a dose-dependent reduction in infarction area after XNJT administration, with a difference of statistically significant in the XNJT-High group (P<0.0001). This suggests a significant protective effect of XNJT on the brain (**Figures 3B, C**). Laser scattering results showed abundant cerebral blood flow in the Sham group and severely diminished blood flow in the left side of the Model group compared to the Sham group (P<0.0001). The treatment groups experienced a rise in brain blood circulation, notably distinct in the XNJT-High group. (P<0.0001) (**Figure 3D, E**).

### 3.3 XNJT attenuates histopathological damage

**Figure 4:**
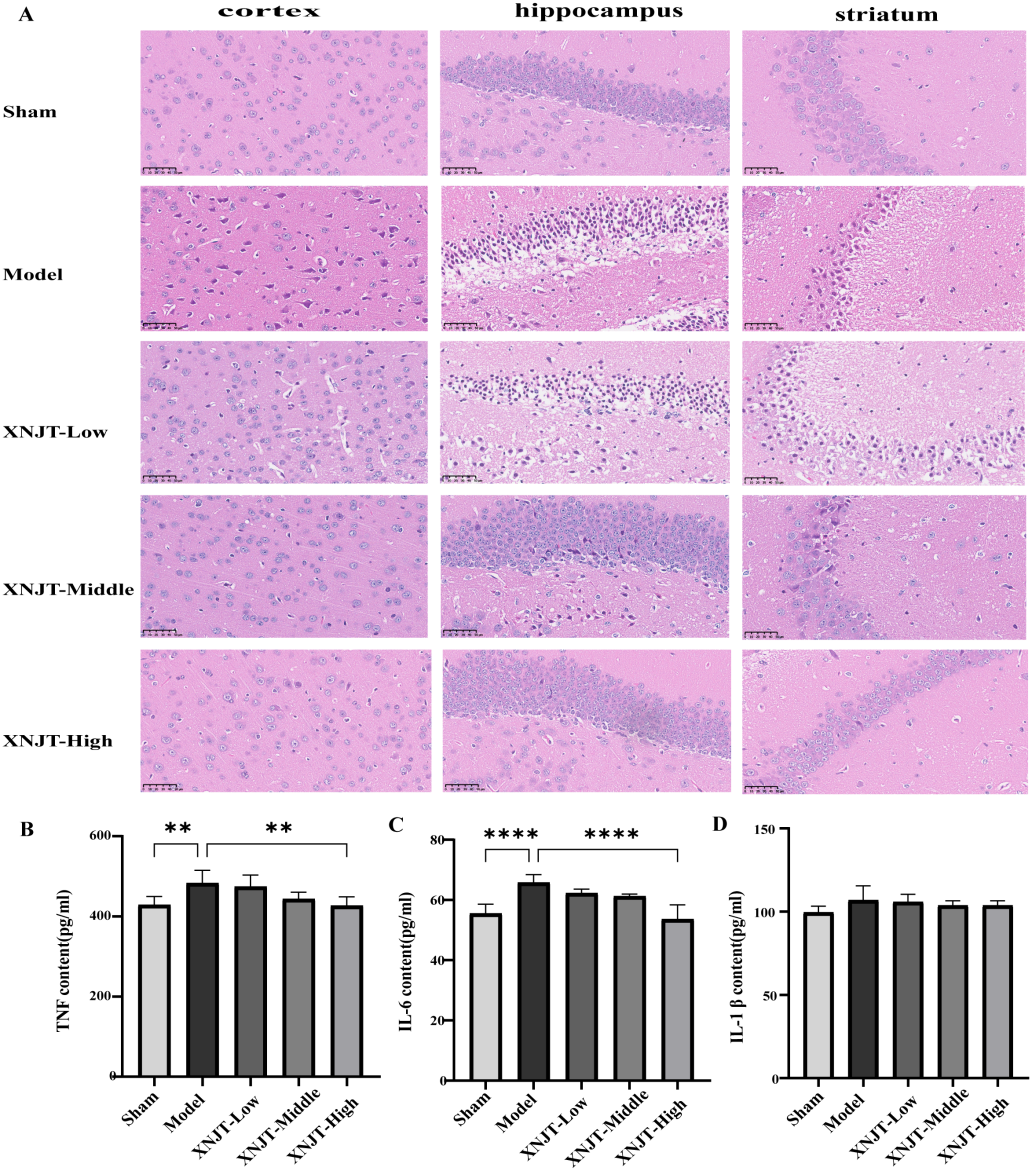
XNJT attenuates histopathological damage and reduces serum levels of inflammatory mediators in mice (n=6). **(A)** The representative images of HE staining. **(B)** TNF serum level. **(C)** IL-6 serum level. **(D)** IL-1b serum level. Statistical differences were examined by one-way ANOVA. **p <0.01, ****p <0.0001.

HE staining showed that the brain tissue of the Sham group was intact, with abundant cells, normal morphology, neat arrangement, intact intercellular structure, tight connections, and normal neurons in the cortex, hippocampus, and striatum. In contrast, the Model group’s mice exhibited numerous deceased neurons, nuclear shrinkage, and vacuoles in the adjacent ischemic cortex, hippocampus, and striatum. Compared to the Model group, improvement levels varied among the treatment groups in the histopathologic features and morphology of the cortex, hippocampus, and striatum, featuring diminished localized necrosis and infiltration of inflammatory cells, along with a more structured cellular configuration (**Figure 4A**).

### 3.4 XNJT reduces serum levels of inflammatory mediators in mice

Compared to the Sham group, the Model group showed elevated levels of TNF, IL-6, and IL-1β (**Figures 4B–D**). In contrast to the Model group, every group undergoing treatment showed reduced levels of TNF, IL-6, and IL-1β. Particularly, the XNJT-High group demonstrated significant differences in TNF (P<0.01) and IL-6 (P<0.0001).

### 3.5 XNJT ameliorates intestinal microbial population dysbiosis

**Figure 5:**
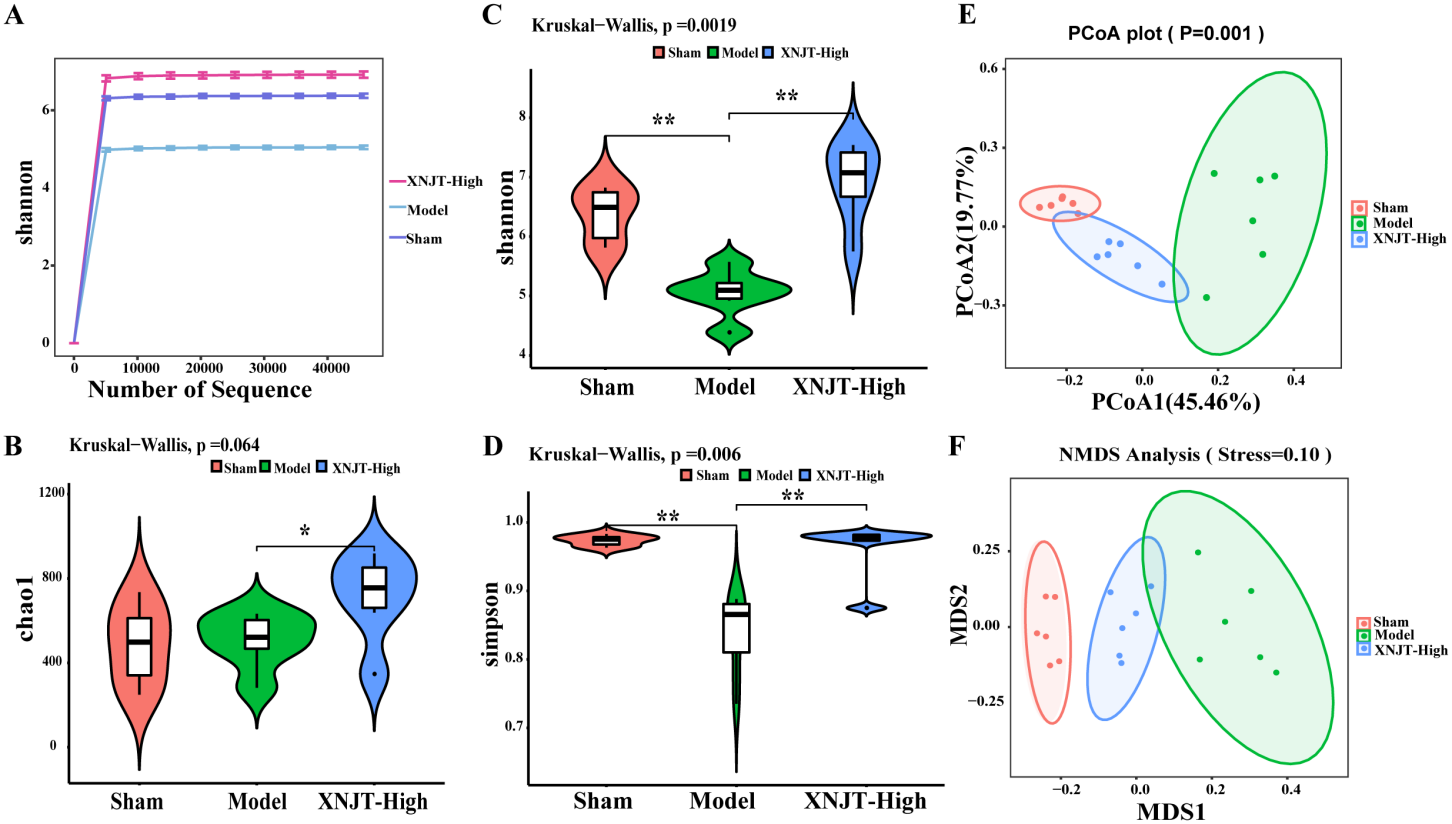
Effect of XNJT on the alpha diversity and beta diversity analysis of the intestinal flora (n=6). **(A)** Rarefaction curve analysis based on the Shannon index. **(B)** Alpha diversity analysis based on the Chao1 index. **(C)** Alpha diversity analysis based on the Shannon index. **(D)** Alpha diversity analysis based on the Simpson index. **(E)** Beta diversity analysis based on the PCoA. **(F)** Beta diversity analysis based on the NMDS. Statistical differences were examined by Kruskal-Wallis test statistic. *p < 0.05, **p <0.01.

In order to confirm our theory that XNJT’s anti-stroke properties are due to altering gut microbiota composition, we conducted 16S rDNA sequencing on the cecum of mice in the Sham, Model, and XNJT-High groups to assess XNJT’s effect on gut microbiota dysbiosis in MCAO/R mice. Alpha diversity analysis reflects species richness, evenness, and sequencing depth. The dilution curve indicates sufficient sequencing depth and sample size (**Figure 5A**). In contrast to the Sham group, the Model group exhibited a notable reduction in both Simpson and Shannon indices (P<0.01). Conversely, the XNJT-High group showed significant rises in Chao1, Shannon, and Simpson indices relative to the Model group (P<0.01) (**Figures 5B-D**). The results indicate that XNJT can influence the diversity of the gut microbiota.

Beta diversity refers to species dissimilarity among different environmental communities. Principal coordinates analysis (PCoA) and Nonmetric Multidimensional Scaling (NMDS) results show that, compared to the Sham group, the Model group exhibited the greatest displacement, while the XNJT-High group was positioned between the Sham and Model groups. This finding suggests that XNJT can regulate intestinal flora dysbiosis in MCAO/R mice (**Figures 5E, F**).

**Figure 6:**
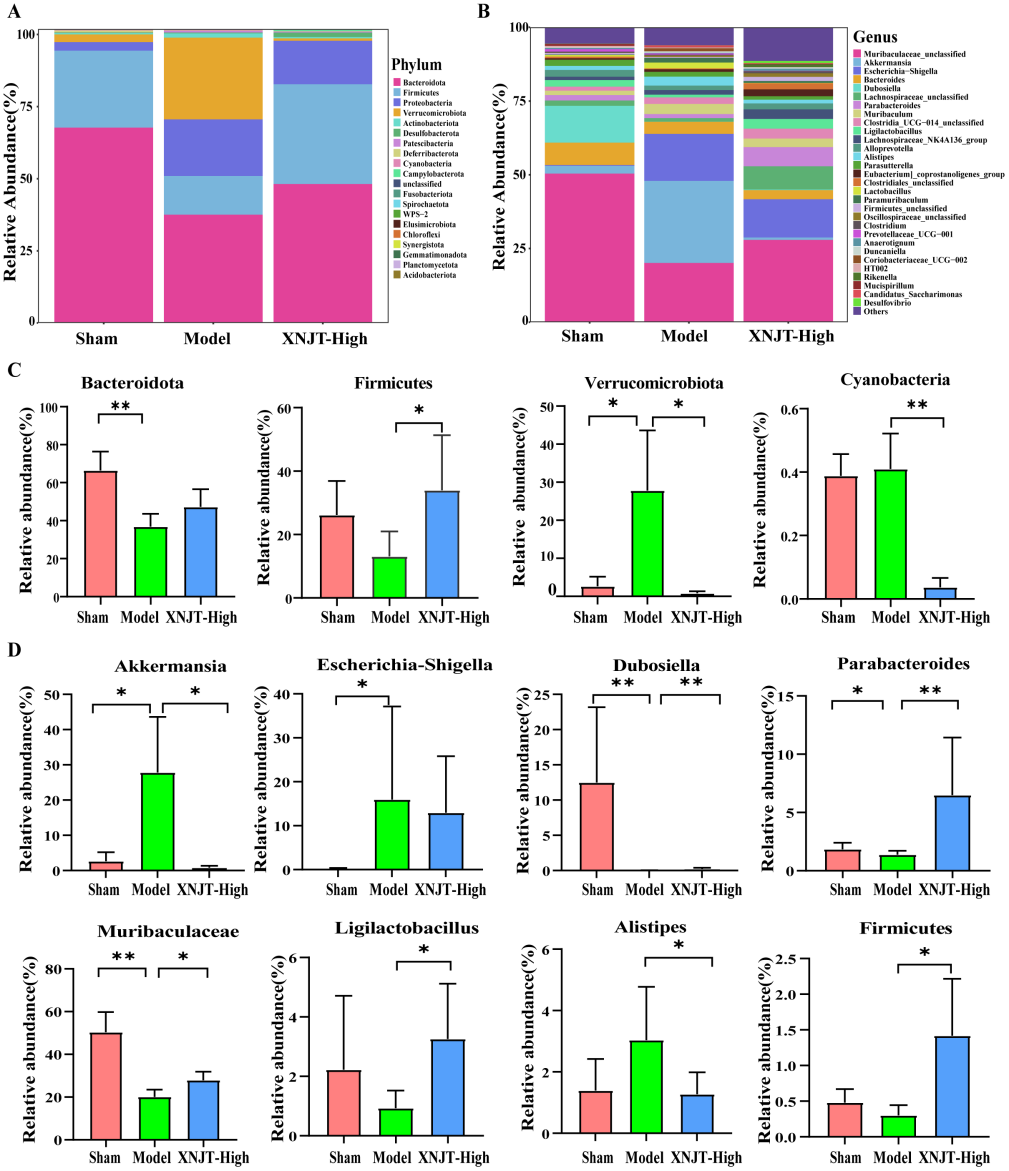
XNJT regulated the gut microbiota at the Phylum and Genus level. **(A)** The gut microbiota composition at the Phylum level. **(B)** The gut microbiota composition at the Genus level. **(C)** The statistical analysis at the Phylum level. **(D)** The statistical analysis at the Genus level. Statistical differences were examined by Kruskal-Wallis test statistic. *p <0.05, **p <0.01.

Differential analysis of microbial abundance at the Phylum and Genus levels revealed significant differences in gut microbiota composition. Although the overall gut microbial community structure among different groups was similar, there were notable variations in the abundance of specific bacteria. At the Phylum level, the Model group showed a decreasing trend in the abundance of *Bacteroidota* and *Firmicutes* compared to the Sham group, while their abundance increased in the XNJT-High group (**Figures 6A, C**). The abundance of *Verrucomicrobiota* and *Cyanobacteria* rose in the Model group but decreased in the XNJT-High group. At the Genus level (**Figures 6B, D**), compared to the Sham group, the Model group exhibited a notable rise in the populations of *Akkermansia*, *Escherichia-Shigella*, and *Alistipes*, alongside a marked reduction in the numbers of *Dubosiella*, *Parabacteroides*, *Muribaculaceae*, *Ligilactobacillus*, and *Firmicutes*. Importantly, the XNJT-High group ameliorated the microbial changes observed at both the Phylum and Genus levels in the Model group, suggesting a significant restorative effect of XNJT on the dysregulated gut microbiota in the stroke model.

**Figure 7:**
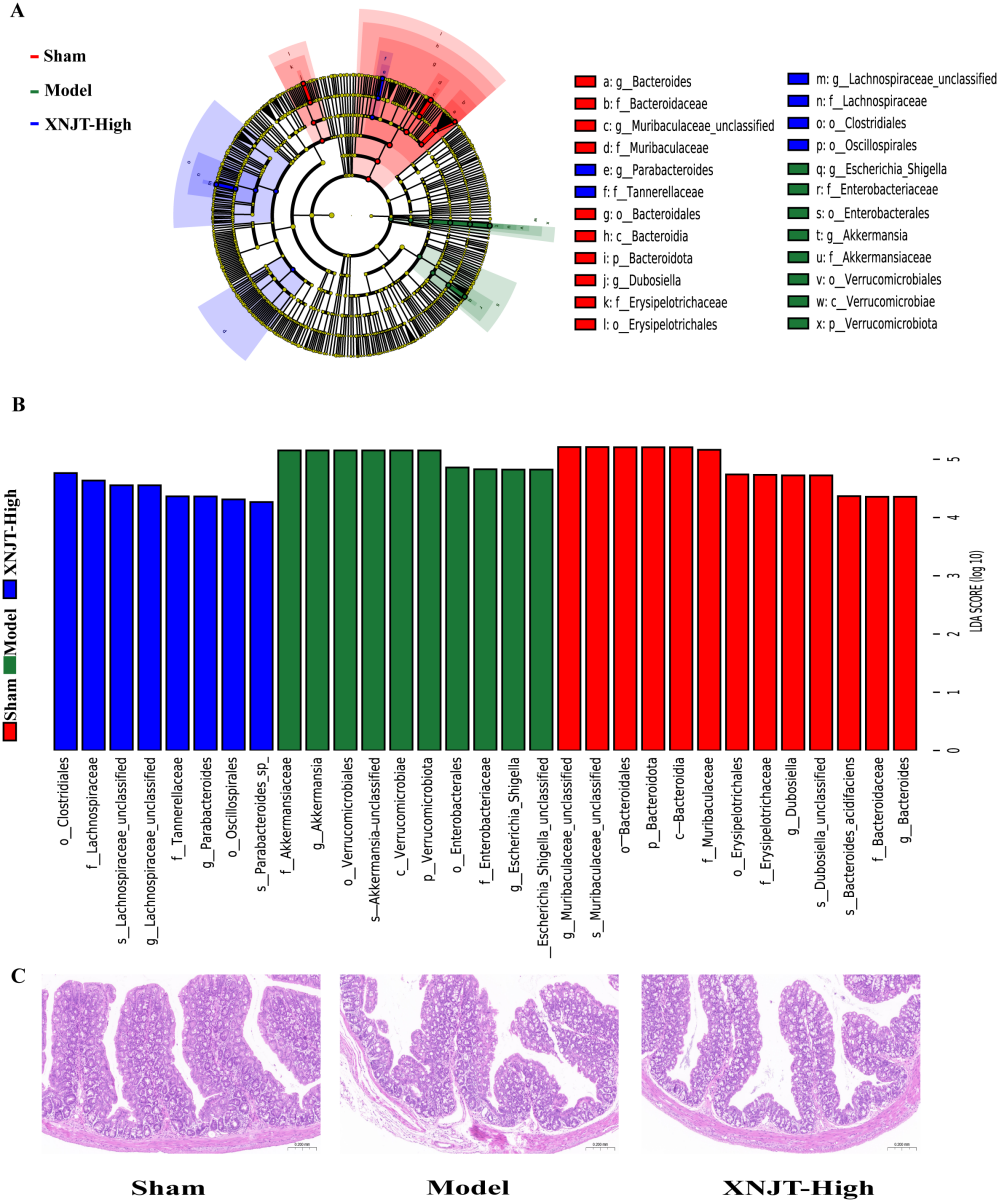
LEfSe analysis of changes in gut microbiota among groups. And HE staining of the intestine. **(A)** Evolutionary branching diagram.The different circle levels in the evolutionary branching diagram radiate from the inside to the outside to represent the seven taxonomic levels of Phylum, Order, Family, Genus, and Species. Each node represents a species classification at that level, and the higher the abundance of the species, the larger the node. The red color of a node indicates that the species is significantly different in the comparison group, and the abundance of the species is higher in the red group. **(B)** LEfSe analysis (LDA>4). The vertical coordinates are the categorical units with significant differences between groups, and the horizontal coordinates visualize the logarithmic score values of the LDA difference analysis for the corresponding categorical units in a bar chart. And the scores are sorted according to the score values to depict the size of their differences in different group samples. The longer the length, the more significant the difference. **(C)** HE staining of the intestine(n=6).

To identify bacteria linked to MCAO/R, we employed Linear discriminant analysis Effect Size (LefSe) analysis to determine specific bacterial differences among the Sham, Model, and XNJT-High groups (**Figures 7A, B**). Our findings demonstrated that the Sham group was enriched with beneficial bacteria such as *Muribaculaceae* and *Dubosiella*. The XNJT-High group exhibited enrichment of beneficial endogenous bacteria such as *Lachnospiraceae* and *Parabacteroides*. In contrast, the Model group mainly enriched *Escherichia-Shigella*. Therefore, the primary bacteria in the Model group could be the ones targeted for the therapeutic effect of XNJT.

The HE staining results showed that the intestinal epithelial cell morphology of mice in the Sham group was as expected, with tightly arranged intestinal glands and a high number of goblet cells. Compared with the Sham group, the intestinal villi in the Model group were loose, the structure of the glands was disorganized and sparsely arranged; the number of goblet cells was reduced; and the muscle layer was damaged and locally necrotic. After treatment with XNJT, a significant improvement could be observed with a positive repair effect (**Figure 7C**).

### 3.6 XNJT influences metabolites in MCAO/R mice cecum contents

**Figure 8:**
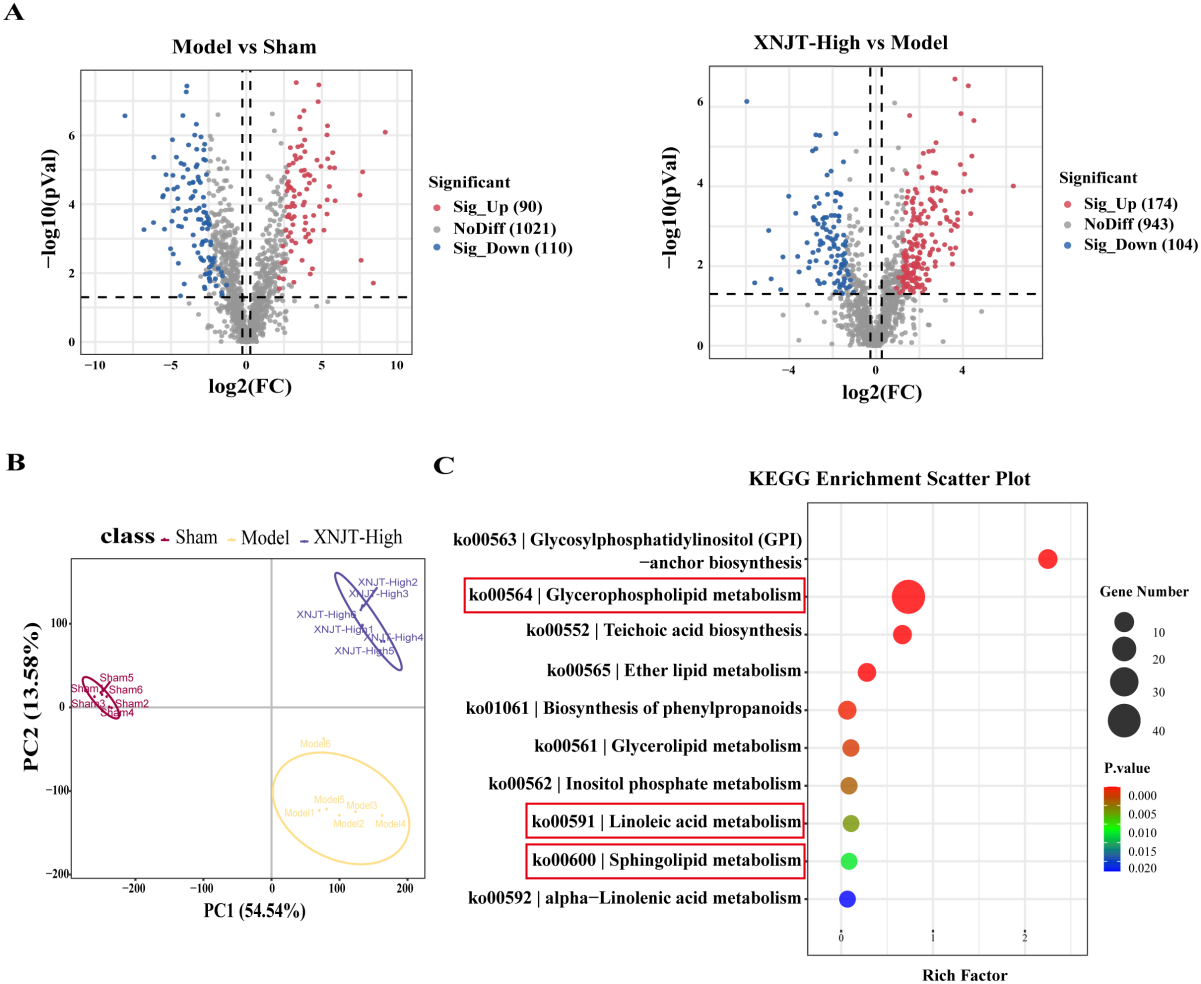
Differences in metabolites between Sham, Model and XNJT-High groups (n=6). **(A)** Volcano graphs. **(B)** PLS-DA scores graph. **(C)** KEGG Enrichment ScatterPlot of differential metabolites.

**Figure 9:**
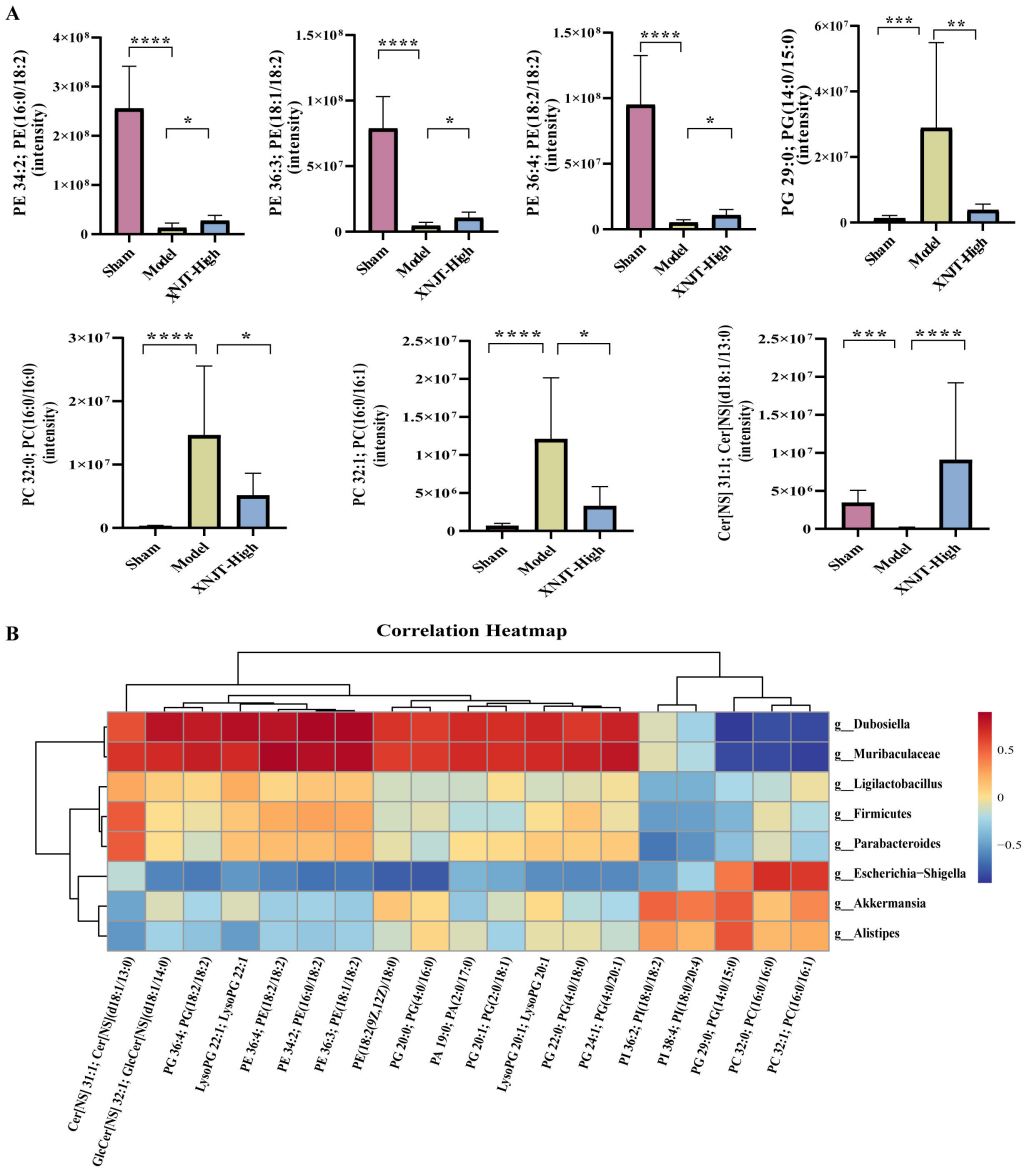
**(A)** Metabolites in the glycerophospholipid metabolism, linoleic acid metabolism, and sphingolipid metabolism pathways. **(B)** The association heatmap of correlation for intestinal flora and metabolites. *p < 0.05, **p <0.01, ***p <0.001, ****p <0.0001.

In the study, a total of 90 upregulated and 110 downregulated metabolites were identified between the Sham and Model groups. Similarly, between the Model and XNJT-High groups, 174 upregulated and 104 downregulated metabolites were found (**Figure 8A**). PLS-DA analysis was used for the exploration of the impacts of XNJT on the metabolites in the cecum contents of mice subjected to MCAO/R (**Figure 8B**). The Sham and Model groups were clearly separated, indicating a significant impact of MCAO/R treatment on metabolite composition. Additionally, a clear distinction was noted between the XNJT-High and Model groups, indicating notable variances in the metabolite profiles of these groups. Statistical analysis showed that 89 metabolites changed significantly during the MCAO/R modeling process. To better understand the efficacy of XNJT, we performed KEGG pathway enrichment analysis (**Figure 8C**). The metabolites were mainly enriched in the following 10 pathways: Glycosylphosphatidylinositol (GPI)-anchor biosynthesis, Glycerophospholipid metabolism, Teichoic acid biosynthesis, Ether lipid metabolism, Biosynthesis of phenylpropanoids, Glycerolipid metabolism, Inositol phosphate metabolism, Linoleic acid metabolism, Sphingolipid metabolism, and alpha-Linolenic acid metabolism. Among them, 19 metabolites showed varying degrees of reverse regulation after drug administration (**Table 2**). **Figure 9A** displayed the comparative signal strengths prevalent across various groups. These metabolites significantly participate in glycerophospholipid metabolism, linoleic acid metabolism, and sphingolipid metabolism pathways.

**Table 2:** Key metabolites enriched in untargeted metabolomics.

Name	Formula	M/Z	RT	P	VIP	Model vs Sham	XNJT-High vs Model	Pathway
PG 20:0; PG(4:0/16:0)	C26H51O10P	553.3124963	3.774866667	0.000007870906539	2.02861446709025	down	up	Glycerophospholipid metabolism
PG 36:4; PG(18:2/18:2)	C42H75O10P	769.4976838	6.052983333	0.000008167201793	1.42084439055549	down	up	Glycerophospholipid metabolism
PE 34:2; PE(16:0/18:2)	C39H74NO8P	714.5047689	6.1197	0.00000002939469	1.30829752426674	down	up	Glycerophospholipid metabolism
PE 36:3; PE(18:1/18:2)	C41H76NO8P	740.5203959	6.195875	0.000000037338253	1.19807647403861	down	up	Glycerophospholipid metabolism
PE 36:4; PE(18:2/18:2)	C41H74NO8P	738.5040334	5.87325	0.000000008669015	1.22913060024248	down	up	Glycerophospholipid metabolism
PG 29:0; PG(14:0/15:0)	C35H69O10P	679.4520047	6.135908333	0.000026954426484	1.07032651616769	up	down	Glycerophospholipid metabolism
PG 20:1; PG(2:0/18:1)	C26H49O10P	551.2965511	3.604	0.000357281438735	1.57760667087623	down	up	Glycerophospholipid metabolism
PG 22:0; PG(4:0/18:0)	C28H55O10P	581.3430365	4.4147	0.000000071418941	2.72719929378525	down	up	Glycerophospholipid metabolism
PG 24:1; PG(4:0/20:1)	C30H57O10P	607.3590102	4.527766667	0.001822881843637	1.72524114413563	down	up	Glycerophospholipid metabolism
PE(18:2(9Z,12Z)/18:0)	C41H78NO8P	742.5360505	6.6191	0.000052799690349	1.17100774761888	down	up	Glycerophospholipid metabolism
PI 38:4; PI(18:0/20:4)	C47H83O13P	885.5451379	6.536533333	0.017611700538597	1.79912573171851	up	down	Glycerophospholipid metabolism
PI 36:2; PI(18:0/18:2)	C45H83O13P	861.5448803	6.60845	0.002493072262281	1.89276443455869	up	down	Glycerophospholipid metabolism
PA 19:0; PA(2:0/17:0)	C22H43O8P	465.2602689	3.111325	0.000000187443306	1.35926007277551	up	down	Glycerophospholipid metabolism
LysoPG 20:1; LysoPG 20:1	C26H51O9P	537.317263	3.987083333	0.00000000327899	1.32448550900654	down	up	Glycerophospholipid metabolism
LysoPG 22:1; LysoPG 22:1	C28H55O9P	565.3484691	4.652433333	0.000392005151984	1.51565028671069	down	up	Glycerophospholipid metabolism
PC 32:0; PC(16:0/16:0)	C40H80NO8P	792.5779317	6.47295	0.000000070811818	1.60681164686669	up	down	Linoleic acid metabolism
PC 32:1; PC(16:0/16:1)	C40H78NO8P	790.5619109	6.1362	0.000023202863808	1.10311516125675	up	down	Linoleic acid metabolism
GlcCer[NS] 32:1; GlcCer[NS](d18:1/14:0)	C38H73NO8	672.5414266	5.645325	0.013282794928206	1.21945948967965	down	up	Sphingolipid metabolism
Cer[NS] 31:1; Cer[NS](d18:1/13:0)	C31H61NO3	496.4728651	7.230208333	0.000003652349275	2.09927136851344	down	up	Sphingolipid metabolism

### 3.7 Spearman correlation analysis

To reveal the interactions among gut microbiota and metabolites, Spearman correlation analysis was performed by us on the 8 Genus levels bacteria and 19 metabolites identified in the study (**Figure 9B**). The results demonstrated significant correlations between gut microbiota and metabolites. Our discovery was that PI 38:4; PI(18:0/20:4), PI 36:2; PI(18:0/18:2), PG 29:0; PG(14:0/15:0), PC 32:0; PC(16:0/16:0), and PC 32:1; PC(16:0/16:1) were positively associated with *Akkermansia* and *Alistipes.* Predict the involvement of *Akkermansia* and *Alistipes* in the synthesis and catabolism of these metabolites. In particular, *Akkermansia* has been associated with a variety of metabolic diseases. These positive correlations suggest that these lipid metabolites may play a role in regulating the growth and activity of these bacteria. However, PI 38:4; PI(18:0/20:4), PI 36:2; PI(18:0/18:2), PG 29:0; PG(14:0/15:0), PC 32:0; PC(16:0/16:0), and PC 32:1; PC(16:0/16:1) were negatively associated with *Firmicutes*, *Ligilactobacillus*, *Muribaculaceae*, *Parabacteroides*, and *Dubosiella*. *Muribaculaceae* play an important role in the production of short-chain fatty acids and have been associated with the development of chronic diseases. This suggests that XNJT treatment of cerebral ischaemia/reperfusion may have a positive impact by modulating the abundance of these bacteria to influence metabolite levels. Additionally, PG(14:0/15:0), PC 32:0; PC(16:0/16:0), and PC 32:1; PC(16:0/16:1) were positively associated with *Escherichia-Shigella*. *Escherichia-Shigella* is associated with intestinal disorders, and this negative correlation suggests that PG(14:0/15:0), PC 32:0; PC(16:0/16:0), and PC 32:1; PC(16:0/16:1) may be key metabolites for XNJT treatment of cerebral ischaemia/reperfusion, and that an increase in these metabolites may have an indirect therapeutic effect on cerebral ischaemia/reperfusion. The involvement of these bacteria in lipid synthesis and catabolism is forecasted.

## 4 Discussion

XNJT’s ability to protect the brain from cerebral infarction could be linked to its influence on inflammation, gut microbiota, and their metabolites. Numerous studies have shown the vital involvement of gut microbiota in the onset of cerebral infarction (Durgan et al., 2019). Through 16S rDNA sequencing, we analyzed the gut microbiota in the Sham, Model, and XNJT-High groups. The results revealed dysregulation in the intestinal microbiota of cerebral infarction mice, with some improvement following XNJT therapy. There was a decrease in gut microbiota alpha diversity in cerebral infarction mice, indicating significant changes in gut microbial composition following cerebral ischemia/reperfusion, consistent with previous studies (Gao et al., 2021). In this study, the enriched phylum in the Model group was mainly *Bacteroidota*. Clinical studies have confirmed a decrease in *Bacteroidota* abundance after ischemic stroke (Yin et al., 2015). Additionally, *Firmicutes* are major phyla of bacteria in mammalian intestines. Studies have shown a negative correlation between *Firmicutes* and the volume of lesion, midline shift, and hemorrhage in ischemic stroke, with a decrease in *Firmicutes* abundance post-stroke (Jeon et al., 2020; Gao et al., 2021; Xian et al., 2022). The research noted a comparable occurrence in the intestinal flora of Model mice, with notable rises in *Bacteroidota* and *Firmicutes* levels post-XNJT therapy, hinting at their potential role in XNJT’s effects against ischemic strokes. At the genus level, our study found a reduction in *Ligilactobacillus* and a rise in *Escherichia-Shigella* in the gut of Model mice, which XNJT could reverse. *Escherichia-Shigella* is a pathogenic bacterium that promotes gut inflammation (Guo et al., 2021). *Ligilactobacillus*, a type of probiotic, plays a role in regulating the immune and barrier functions of the intestines, enhancing permeability, reducing inflammation, and lowering serum levels of IL-6, IL-1β, and TNF (Waitayangkoon et al., 2020). Taking *Ligilactobacillus* supplements has been demonstrated to boost cognitive abilities, elevate mood, and reduce inflammation related to aging (Chen et al., 2019c). This suggests that *Escherichia-Shigella* and *Ligilactobacillus* may be crucial microbial species in the XNJT-mediated anti-ischemic stroke inflammatory response.

The severity of ischemic stroke is not only linked to dysbiosis of intestinal microbiota but may also be closely related to metabolites. A comprehensive study suggests that stroke may increase the permeability of the intestines, stimulate the immune response, and exacerbate ischemia/reperfusion injury via the gut-brain pathway (Hu et al., 2022). Importantly, some metabolites produced by gut microbiota through the gut-brain axis inhibit post-stroke inflammation and promote neural function repair, thereby alleviating ischemia/reperfusion injury (Zhao et al., 2024). Research has shown that cerebral ischemic stroke leads to neurological damage and alters histomorphological structures, while also inducing systemic issues such as an imbalanced gut microbiota and increased intestinal permeability (Chen et al., 2019a). When the intestinal barrier’s function is compromised, intestinal bacteria can trigger a systemic inflammatory response, potentially becoming life-threatening (Feng Zhang and Wu, 2020). Our study found loose intestinal villi, disorganised glandular structure, damaged muscularis propria and localised necrosis in mice with cerebral ischaemia/reperfusion model. There was an improvement after using XNJT. *Ligilactobacillus* modulates tight junction proteins, exerting a protective influence on the epithelial barrier (Karczewski et al., 2010). Based on both their research findings and our experimental data, *Ligilactobacillus* dysregulation within the intestine following a stroke may be a contributing factor to intestinal barrier disruption. Therefore, addressing intestinal damage can positively influence blood-brain barrier function and facilitate neurological recovery following a stroke. Previous studies have reported that cerebral ischemia-hypoxia causes physical harm to brain tissue, affecting its biochemical and metabolic functions, and leading to abnormal fluctuations in metabolite levels such as lipids, fatty acids, and amino acids, consistent with our findings (Yang et al., 2022b). Through this research, we obtained the metabolic profiles of gut contents from Sham, Model, and XNJT-High groups through untargeted metabolomics and found metabolic dysregulation in cerebral infarction mice. KEGG enrichment analysis revealed that Glycerophospholipid, Linoleic acid, and Sphingolipid metabolism were predominantly concentrated within these groups. From a metabolomics perspective, these changes constitute the metabolic characteristics of a stroke.

*In vivo* studies have shown that a strong correlation between irregular lipid metabolism and the prediction and prognosis of brain infarction (Liu et al., 2021; Qian et al., 2023). Various research findings suggest that lipids are vital in the occurrence and advancement of illnesses. Compared to proteins, lipids traverse the blood-brain barrier with greater ease, and the brain’s rich presence of polyunsaturated fatty acids renders it more vulnerable to oxidative stress than many other tissues and organs (Hamilton et al., 2007). Lipids include phospholipids, sphingolipids, and glycerides. The primary constituents of biological membranes, phospholipids, are glycerophospholipids and sphingophospholipids, playing a role in signal transduction (Farooqui et al., 2007). Glycerophospholipids undergo metabolic processes in various life stages, categorized into phosphatidyl cholines (PC), phosphatidyl ethanolamines (PE), phosphatidyl inositols (PI), phosphatidyl glycerol (PG), etc., based on their biological functions. Alterations in their composition may impact the stability, permeability, and fluidity of neuronal cell membranes, potentially resulting in neurological disorders (Liu et al., 2024). Interfering with the metabolism of glycerophospholipids usually leads to the swift production and build-up of free fatty acids and lyso-phospholipids (Yang et al., 2022a). In this study, metabolites such as PI 36:2; PI(18:0/18:2), PG 29:0; PG(14:0/15:0), PC 32:0; PC(16:0/16:0), and PC 32:1; PC(16:0/16:1) were notable rise in all of these in the Model group when contrasted with the Sham mice. Notably, the alterations in the previously mentioned metabolites in MCAO/R mice showed substantial improvement following treatment with XNJT. The findings highlighted the critical roles of Glycerophospholipid, Linoleic acid, and Sphingolipid metabolism in cerebral ischemia/reperfusion damage, and also suggested XNJT’s positive impact on MCAO/R mice.

Additionally, our research focused on exploring the link between intestinal microbiota and metabolites found in feces. The examination revealed that *Firmicutes* and *Ligilactobacillus* were negatively correlated with PC and PE levels in the MCAO/R mice. Given the substantial rise in these metabolite levels within the Model group, a trend that was reversed after administration of XNJT, they may be risk factors for cerebral ischemia/reperfusion. Additionally, *Akkermansia* and *Escherichia-Shigella* were positively correlated with PG and PC. In summary, These findings indicate that XNJT could potentially alter gut microbiota composition, thereby affecting fecal metabolite levels. These metabolite alterations are expected to affect specific metabolic pathways that ultimately contribute to the therapeutic effects of XNJT.

Our current study has the following limitations: we exclusively utilized male mice to investigate the effects of XNJT on neurological performance and cerebral stroke. This does not take into account potential gender differences in treatment response. This may also restrict the generalizability of our findings. Therefore, future studies should incorporate research on female mice to ascertain whether there are sex-specific responses to XNJT and to better understand the underlying mechanisms of these potential differences. By doing so, we can enhance the applicability of our research outcomes and contribute to a more comprehensive understanding of the effects of XNJT within a broader context. In addition, our study focused on neurobehavioural scores at 24 hours of MCAO/R and did not perform long-term behavioural monitoring. Since neurobehavioural changes in ischemic stroke can last for several days, we consider performing long-term neurobehavioural scoring in future studies to observe changes in mice during different periods of time.

## 5 Conclusion

In this study, we employed a combination of 16S rDNA sequencing and untargeted metabolomics to investigate the therapeutic effects of XNJT on MCAO/R model mice. Our central finding is that XNJT significantly ameliorates cerebral ischemia/reperfusion injury in MCAO/R mice, reduces levels of inflammatory factors, and modulates gut microbiota dysbiosis and metabolic differences. Notably, the regulatory effects of XNJT on specific bacteria such as Bacteroides, Firmicutes, Escherichia-Shigella, and Ligilactobacillus, as well as its impact on metabolic pathways of glycerophospholipids, linoleic acid metabolism, and sphingolipid metabolism, provide a scientific basis for the efficacy of XNJT. In summary, the imbalance of gut microbial ecology in MCAO/R model mice is closely related to the prognosis of stroke, and XNJT effectively enhances the prospects of stroke treatment by improving intestinal barrier function and modulating gut microbiota and their metabolites.This discovery offers a new perspective for clinical drug intervention in the treatment of cerebral ischemia/reperfusion injury, especially in utilizing the gut microbiota as a therapeutic target, and XNJT may become an innovative approach for traditional Chinese medicine in treating cardiovascular and cerebrovascular diseases.

Moving forward, we need to further investigate the specific regulatory mechanisms of XNJT on the gut microbiota. This includes its direct and indirect effects on specific bacterial communities and how these changes affect the host’s immune response and metabolic status. Additional studies using fecal microbiota transplantation as an auxiliary method are anticipated to uncover the microbial mechanisms by which XNJT improves stroke, providing new insights for the clinical treatment of ischemic stroke.

## Data Availability

The datasets presented in this study can be found in online repositories. The names of the repository/repositories and accession number(s) can be found below: BioProject: PRJNA1191991.
